# Poly lactic-co‐glycolic acid enhances the efficacy of the phytomedicine chrysin against cisplatin induced toxicity in submandibular salivary glands

**DOI:** 10.1038/s41598-025-93112-3

**Published:** 2025-03-25

**Authors:** Samah K. Ezzat, Hend Mohamed Anter, Ola A. Habotta, Doaa A. M. Esmaeil, Doaa A. Farag, Reham Mokhtar Aman

**Affiliations:** 1 Department of Oral Biology, Faculty of Dentistry, Mansoura University, Mansoura, Dakahlia 35516 Egypt; 2 Department of Pharmaceutics, Faculty of Pharmacy, Mansoura University, Mansoura, Dakahlia 35516 Egypt; 3 Department of forensic medicine and toxicology, Faculty of Veterinary Medicine, Mansoura University, Mansoura, Dakahlia 35516 Egypt; 4 Department of Oral Pathology, Faculty of Dentistry, Mansoura University, Mansoura, Dakahlia 35516 Egypt; 5 Department of Oral Pathology, Faculty of Dentistry, Sinai University, Kantra Campus, Ismaeli, Egypt

**Keywords:** Chrysin, Poly(d, L-lactide-co-glycolide) nanoparticles, Cisplatin-induced toxicity, Submandibular salivary glands., Biophysics, Cancer, Drug discovery, Molecular biology, Structural biology, Diseases, Molecular medicine, Oncology, Nanoscience and technology

## Abstract

Chemotherapy, particularly cisplatin, is a prevalent cancer treatment. Unfortunately, many tissues, for instance the submandibular salivary glands, are toxically affected by cisplatin. Of significant interest, phytopharmaceuticals rich in flavonoids have demonstrated exceptional defense against chemotherapy induced toxicity, like chrysin (Chr); nevertheless, its low solubility and poor bioavailability have remained cornerstone issues. Accordingly, Chr was successfully encapsulated in the poly(d, l-lactide-co-glycolide) nanoparticles (PLGA NPs) scaffold. The developed chrysin-loaded poly(d, l-lactide-co-glycolide) nanoparticles (Chr-loaded PLGA NPs) were meticulously evaluated via comprehensive in vitro-in vivo investigations. Saliently, the outcomes of in vivo studies exhibited admirable in vivo counteraction effectiveness against cisplatin-induced toxicity in submandibular salivary glands in Albino rats upon comparing Chr-loaded PLGA NPs treated group with pure Chr as well as blank NPs treated ones. Inclusively, Chr-loaded PLGA NPs can be regarded as promising therapy to create recent vistas for dampening myriad adverse effects of different chemotherapies.

## Introduction

Globally, cancer is one of the leading causes of mortality^1^. Chemotherapy, as the principal nonsurgical remedy for cancer treatment, is often based on the cytotoxic impact of synthetic chemicals, taking advantage of the higher vulnerability of cancer cells as compared to normal ones^2^. Cisplatin and its derivatives are commonly used chemotherapeutic drugs for the treatment of numerous forms of cancer, but they have serious side effects namely; nephrotoxicity, ototoxicity, neurotoxicity, cardiotoxicity, hepatotoxicity, gastrointestinal toxicity, and hematopoietic system injury, which often hinder their clinical uses^3^. Additionally, they decrease salivary glands’ function and raise the risk of mucositis^2,4,5^. Therefore, it is urgently needed to develop effective management strategies against chemotherapy-induced side effects.

**Fig. 1:**
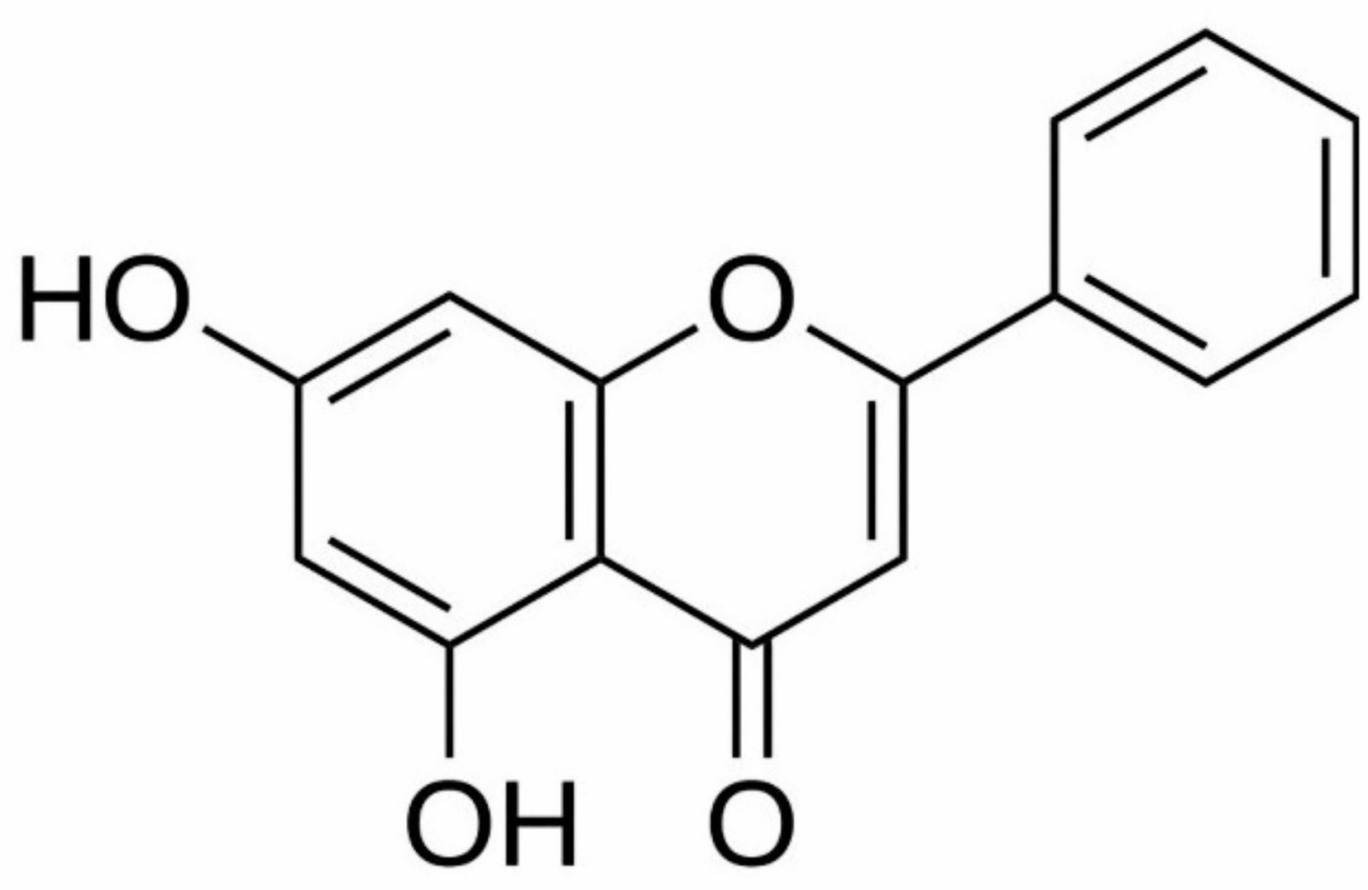
Structural formula of Chr. Abbreviations: Chr; chrysin.

Phytopharmaceuticals are plant-based natural chemicals that have multiple pharmacological effects against various ailments, unlike synthetic drugs^6^. Among the naturally occurring polyphenolic phytochemicals, that are well-known for their valuable properties for human health, is chrysin (Chr). Chr (5,7-dihydroxyflavone, Fig. 1) is a flavonoid with a naturally occurring 15-carbon backbone. Honey, propolis, mushrooms, stingless bee products, and a variety of plant species are the most prevalent naturally occurring sources of Chr^7^. Like various native polyphenolic phytochemicals, it owns prominent antioxidant, anti-inflammatory, anti-neoplastic, antiviral, antidiabetic, and neuroprotective activities which have been proved in a variety of animal models and cell lines^8–14^. Chr belongs to class II of the biopharmaceutical categorization system (BCS)^15^. As a consequence, its limited water solubility significantly lowers its bioavailability and limits its application^16^. Therefore, the use of Chr in appropriate delivery systems has been offered as a solution to overcome these challenges and to enhance Chr’s bioactivities^15,17–20^.

Polymeric nanoparticulate delivery systems (PNDS) are nanoscale solid particles or dispersions generally prepared from biocompatible and biodegradable natural or synthetic polymers. For the incorporation of plant-based natural chemicals into PNDS, the most widely used synthetic polymers, in a variety of composition ratios, are poly(d, l-lactide-co-glycolide) (PLGA), poly(d, l-lactic acid) (PLA), and polycaprolactone^21^.

PLGA is one of the most prevalent polymers adopted in the formulation of drug delivery systems, for biomedical applications, owing to its biodegradability, biosafety, and biocompatibility. Additionally, it has been approved by the United States Food and Drug Administration (US FDA) for human use in nanoformulations. Poly(d, l-lactide-co-glycolide) nanoparticles (PLGA NPs), as one of the PNDS, improve medication bioavailability by preventing early breakdown in biological fluids and allowing for targeted administration, enhanced bioactive ingredient penetration as well as reduced side effects^22–24^. Although documented lines of substantiation manifesting Chr’s efficacy against chemotherapy induced toxicity in various organs have been demonstrated, no nanostructured delivery system has been developed to investigate its effective counteraction in cisplatin-induced toxicity in submandibular salivary glands.

Thereupon, the aim of the current study to develop an injectable chrysin-loaded poly(d, l-lactide-co-glycolide) nanoparticles (Chr-loaded PLGA NPs) to explore its effective counteraction against cisplatin-induced toxicity in submandibular salivary glands in Albino rats. Accordingly, Chr was entrapped in the PLGA NPs scaffold by a double emulsion/solvent evaporation technique (w_1_/o/w_2_). The developed Chr-loaded PLGA NPs was appraised regarding its drug entrapment efficiency (DEE%), particle diameter (D_p_), polydispersity index (PI), zeta potential (ζP), morphological analysis, Fourier transform-infrared (FT-IR) spectroscopy, differential scanning calorimetry (DSC), x-ray diffraction (XRD), in vitro release study, physical stability, and ultimately exploring the in vivo efficacy against cisplatin-induced toxicity in submandibular salivary glands in Albino rats.

## Materials and methods

### Materials

Chr (CAS Number: 480-40-0, Lot: 10224507) was purchased from Thermo Fisher ((Kandel) GmbH, Erlenbachweg 2, 76870 Kandel, Germany). Methanol (high performance liquid chromatography (HPLC)-grade, Fischer) was procured from Cornell lab (Cairo, Egypt). PLGA, carboxylic acid-terminated copolymer, glycolide: lactide ratio of 50:50; PURASORB^®^ PDLG 5002 A] was graciously supplied via Corbion Purac Biomaterials (Amsterdam, Netherlands). Polyvinyl alcohol (PVA) with a molecular weight (M_w_) of 31–50 KDa was acquired from Acros organics (Geel, Antwerp, Belgium). Dimethyl sulfoxide (DMSO), dichloromethane (DCM), tween 80, disodium hydrogen phosphate (Na_2_HPO_4_), potassium dihydrogen orthophosphate (KH_2_PO_4_), absolute ethanol, propylene glycol-200 (PG-200), and sodium carboxymethylcellulose (sodium CMC) were obtained from Adwic, EL Nasr Pharmaceutical Chemicals Co. (Cairo, Egypt).

### Preparation of Chr-loaded PLGA NPs

Chr-loaded PLGA NPs formulae were developed adopting double emulsion/solvent evaporation technique (w_1_/o/w_2_) (F1 to F4, Table 1), as previously represented, with minor amendments^25,26^. Initially, Chr (Chr_total_) and PLGA, in a weight ratio of either 1:10 or 1:15, respectively, were dissolved in 5 mL of the organic solvent mixture, DCM/methanol in a ratio of 3:2 v/v “organic phase (o)”, using a vortex mixer (Model VM-300, Gemmy Industrial Corp., Taiwan).

In an ice bath, 2.5 mL of PVA (0.5% w/v), as the internal aqueous phase (w_1_), were dropped on the organic phase (o) and directly sonicated (Model VC 505, Sonics Vibra-cell™, Sonic & Materials, Inc., USA) whilst complying the following conditions: (Timer: 60 s, Amplitude: 90%, Pulser: 1 s ON/1 second OFF). Thereupon, to prepare the double emulsion (w_1_/o/w_2_), the outcome primary emulsion (w_1_/o) was rapidly diffused into the external aqueous phase (w_2_), 15 mL PVA (0.5 or 1% w/v), and sonication procedure was repeated adopting the identical conditions for additional 60 s. The organic solvent mixture evaporation was implemented via continuous magnetic stirring nightlong (500 rpm) at ambient conditions.

Utilizing the cooling centrifuge (CE16-4 × 100RD, ACCULAB, USA), the un-encapsulated Chr (Chr_free_) was isolated from the developed Chr-loaded PLGA NPs at 13,000 rpm and 4 °C for 2 h. The assembled supernatant, including Chr_free_, would be maintained to estimate the DEE %, whilst the gathered NPs were washed with purified water (PW), redispersed in PW and freeze-dried by freeze dryer (SIM international, SIM FD8-8 T, USA). At last, the freeze-dried Chr-loaded PLGA NPs were conserved at 4 °C for forthcoming investigations. An identical procedure was employed to prepare the analogous empty PLGA NPs formulae (blank NPs) without Chr in the organic phase (o).

**Table 1:** Composition of Chr-loaded PLGA NPs.

Formula code	Chr/PLGA (w/w ratio)	PVA concentration (% w/v)
F1	1:10	1
F2	1:15	1
F3	1:10	0.5
F4	1:15	0.5

Abbreviations: Chr-loaded PLGA NPs; chrysin-loaded poly(d, l-lactide-co-glycolide) nanoparticles, Chr; chrysin, PLGA; poly(d, l-lactide-co-glycolide), PVA; polyvinyl alcohol.

### Characterization of Chr-loaded PLGA NPs

#### Determination of DEE %

To determine DEE % for all the prepared formulae, an indirect method was adopted. After cooling centrifugation, Chr_free_, in the assembled supernatant, was estimated by ultraviolet/visible (UV–VIS) spectroscopy (JENWAY 6850, UV–VIS double beam spectrophotometer, UK), against the supernatant of blank NPs analogous to each formula, at λ_max_ 339 nm^27^. DEE % was calculated for each formula using Eq. (1) ^27^:

1 $${\text{DEE\% }}=\left[ {{\text{Chr in NPs}}} \right.\left( {{\text{Ch}}{{\text{r}}_{{\text{total}} }}}- \right.\left. {\left. {{\text{Ch}}{{\text{r}}_{free}}} \right)} \right]/\left. {{\text{Ch}}{{\text{r}}_{{\text{total}}}}} \right]~ \times 100$$

#### Determination of Dp, PI and ΖP

For all the freshly prepared Chr-loaded PLGA NPs formulae, after suitable dilution with PW, the mean D_p_, PI and ζP were appraised utilizing a Zetasizer Nano ZS apparatus (Malvern Instruments, Malvern, UK). Dynamic light scattering (DLS) as well as Laser Doppler Electrophoresis (LDE) techniques were operated, by the utilized apparatus, to measure the average D_p_ and ζP, respectively.

### Characterization of the chosen formula

#### Morphological analysis

Permanently, the NPs’ morphological features attain considerable attention as the morphology always affects most of the NPs’ properties. Transmission electron microscopy (TEM), based on electron transmittance principle, is one of the most important utilized microscopic techniques for morphological analysis. The morphology of the chosen PLGA NPs formula loaded with Chr was studied via this technique (TEM, JEOL JEM-2100, JEOL Ltd., Tokyo, Japan). Following reasonable dilution of NPs colloidal dispersion with PW and sonication, just a drop of the diluted dispersion was loaded on a grid “carbon-coated copper one”. Afterwards, the excess sample was drained by filter paper, the grid was allowed for dehydration at ambient temperature, and ultimately the sample was inspected via TEM instantly, with no staining. The image capture and analysis procedure, including particle size, were accomplished via Digital Micrograph and Soft Imaging Viewer software.

#### FT-IR spectroscopic analysis

FT-IR, a quick, easy, and economical technique, is widely used for identifying the functional groups of an extensive range of substances including NPs as well as their constitutive ingredients. The infrared spectra of Chr, PLGA, PVA, the comparable physical mixture (PM) with the analogous ratio utilized throughout the formulation of the chosen PLGA NPs formula, besides freeze-dried blank and medicated PLGA NPs, were individually analyzed using a FT-IR Spectrophotometer (Bruker Alpha II Platinum, Billerica, MA 01821, USA). Samples were scanned over a range of 500–4000 cm^− 1^, employing the Attenuated Total internal Reflectance (ATR) technique.

#### DSC analysis

DSC is a thermoanalytical technique utilized to recognize thermal transitions of NPs constituents during the process of NPs formulation. The calorimetric behavior of Chr, PLGA, PVA, the comparable PM with the analogous ratio utilized throughout the formulation of the chosen PLGA NPs formula, besides freeze-dried blank and medicated PLGA NPs, was outlined using a DSC thermal analyzer (LABSYS evo TG-DTA/DSC, Setaram Corp., Caluire, France) calibrated with indium as the reference standard. The next conditions namely; Sample pan: hermetically closed aluminum pan, Sample weight: 5 mg, Heating rate: 10 °C/min, Temperature scanning range: from 30 to 400 °C, Nitrogen flow rate: 25 mL/min, were chosen to be followed.

#### XRD analysis

XRD is one of the most important characterization techniques used to provide information regarding the crystalline, semicrystalline, or amorphous structure of the NPs, besides their native constituents. XRD patterns of Chr, PLGA, PVA, the comparable PM with the analogous ratio utilized throughout the formulation of the chosen PLGA NPs formula, besides freeze-dried blank and medicated PLGA NPs, were assessed adopting a Diano X-ray diffractometer (USA). The next conditions namely; Voltage: 45 kV, Current: 9 mA, Scanning range: from 3° to 50° at 2θ angle, were obeyed.

#### In vitro release study

To inspect the in vitro release profile of Chr from the freshly-made chosen PLGA NPs formula loaded with Chr and compare it with its diffusion profile from aqueous suspension (as a control), vertical Franz diffusion cells, designed locally with a 3 cm diameter, were used.

In brief, before fixation amid the donor compartments and receptor ones, the cellulose membranes (Spectra/Por™, Spectrum Medical Industries Inc., M_w_ cut off: 12–14 KDa, Los Angeles 90054, USA) were kept in equilibrium with phosphate buffer (PB, pH 7.4), as the release medium, for 12 h. Then, the receptor compartments were filled with 100 mL of PB (pH 7.4) containing tween 80 (co-solvent, 1%, v/v), as the release medium^28^, while the donor ones were filled with colloidal dispersion of the chosen PLGA NPs formula containing an equivalent amount of 2 mg Chr.

The diffusion cells were continuously shaked by thermostatically controlled shaking incubator (GFL Gesellschaft für Labortechnik, Burgwedel, Germany) kept-up at a temperature of 37 ± 0.5 °C and a speed of 100 rpm. At predetermined time intervals; 0.5, 1, 2, 3, 4, 6, 8, 10, 24, 48 and 72 h, a sample was taken out of the receptor compartment (3 mL) then replaced by an equivalent volume of the fresh release medium, kept at 37 ± 0.5 °C, to keep the release medium’s volume constant during the investigation. Hereupon, the cumulated samples were analyzed spectrophotometrically for Chr concentration using UV–VIS spectrophotometer, at λ_max_ 272 nm, against the corresponding blank NPs for chosen PLGA NPs formula which was handled similarly as the medicated one. The cumulative Chr released (%), at each time interval, was calculated as a mean of three trials. Comparably, for the diffusion process of Chr, an aqueous suspension containing equivalent quantity of pure Chr was also investigated, in triplicate.

#### Kinetic analysis of Chr release data

The mechanism of Chr release from the chosen PLGA NPs formula as well as pure Chr in the PB, as the release medium, was determined via fitting the obtained in vitro release data into different mathematical kinetic models, including zero-order, first-order, and Higuchi^29^. Furthermore, to demonstrate the release mechanism, Korsmeyer–Peppas kinetic model was also applied^30^. The kinetic model which gives coefficient of determination (R^2^) value close to 1 would be chosen as the one that best described the release profile.

#### Stability study

As previously reported, the stability study of the chosen PLGA NPs formula loaded with Chr was evaluated^31^. Colloidal dispersions, of the chosen formula, were prepared and stored at refrigerator (4 ± 1 °C) for 3 months. NPs were examined regarding D_p_ and PI at 0, 1, 2 and 3 months^32^. Additionally, the average drug retention percent (ADR%) was also calculated over the storage period, as follows in Eq. (2):

2 $$~{\text{ADR}}\% ~ = ~\frac{{EE~\% ~\;{\text{at}}\;{\text{each}}\;{\text{time}}\;{\text{interval}}}}{{~{\text{initial}}~{\text{EE}}\% }} \times 100$$

### Effective counteraction of the chosen Chr-loaded PLGA NPs formula against cisplatin-induced toxicity in submandibular salivary glands in Albino rats

#### Study design

The sample size was calculated using G*Power 3.1.9.2. In a one-way analysis of variance (ANOVA) study, a sample size of forty rats (eight in each group) using the F test (ANOVA: fixed effects, omnibus, one way) with a 0.05 alpha significance level and 0.8 power to achieve a large effect size of 0.6.

#### Animals

Upon arrival, adult male Albino rats (Egyptian Organization for Biological Products and Vaccines Giza, Egypt), two months old and weighing 100 to 150 gm were housed in individual cages and kept in 12/12 dark and light cycle with relative 50–70% humidity. Rats were acclimatized for one week before the experiments and provided free access to standard laboratory animal diet and water. Regarding the use and care of animals, the Research Ethics Committee, Sinai University (SU.REC.2024(4 A)), approved this study (related Files ). This study followed the guidelines of the Animal Research: Reporting In vivo Experiments (ARRIVE) as well as their checklist. All procedures followed in accordance with the American Veterinary Medical Association’s guidelines. ( https://www.nc3rs.org.uk/arrive-guidlines )

#### Experimental outline

To reconnoiter the chosen Chr-loaded PLGA NPs formula’s ability to dampen cisplatin-induced toxicity in submandibular salivary glands in Albino rats, cisplatin was first intraperitoneally (IP) injected to most of the used experimental rats “thirty-two out of forty adult male Albino ones” with a daily dose of 5 mg/kg and for 3 consecutive days (total dose of cisplatin 15 mg/kg)^33^. Then, they were randomly allocated (using random number tables), divided into five groups (eight rats per group) and given treatments as follows:

- Group A: Negative control (N); rats did not receive cisplatin nor any treatment.
- Group B: Cisplatin-induced toxicity; rats received no treatment after IP injection of cisplatin.
- Group C: Pure Chr; rats received pure Chr suspension (50 mg/kg suspended in 1% w/v sodium CMC), 5 times per week for 3 weeks by oral gavage tube^17^.
- Group D: Blank NPs; rats were intravenously (IV) injected with blank NPs suspension (unloaded with Chr) equivalent to the chosen Chr-loaded formula, 3 times per week for 3 weeks.
- Group E: Chr-loaded PLGA NPs; rats were treated as similar to group D but with the chosen Chr-loaded PLGA NPs formula (IV, 5 mg/kg)^17^.

Pure Chr solution in a mixture of DMSO, PG-200, normal saline, and absolute ethanol at a ratio of 3:3:2:2, respectively (IV, 5 mg/kg)^34^, was tested and the obtained devastating effects on the rats’ tails prevented the researchers from completing the experiment (data not shown).

#### Histopathological and immunohistochemical (IHC) staining

After the end of the experiment, the rats were euthanized using an intraperitoneal injection of 200 mg/kg sodium pentobarbital euthanasia solution. Submandibular salivary glands were excised and prepared into paraffin blocks. In brief, the animal specimens were kept in 10% formaldehyde solution for 24 h. After fixation, the specimens were scrubbed under running water to remove residual fixatives. The specimens were dried in a series of successive alcohol baths before being cleared with xylene, and tissue slices were prepared using an automated tissue processor.

The infiltrated specimens were embedded within melted paraffin. Cutting was done at a thickness of 4–6 μm using a microtome. Hematoxylin and eosin (H&E) staining was applied to the obtained sections as a routine one, besides, IHC staining for monoclonal anti-cleaved caspase-3 (Asp 175; 1:2000) was also employed. Every section was evaluated blindly by two histopathologists.

#### Real time-polymerase chain reaction (RT-PCR) analysis

Salivary gland samples for the gene expression study were snap-frozen and kept in liquid nitrogen until the RNA extraction procedure. Tissue samples, weighing 10 mg each, were homogenized using a Kinematica Polytron homogenizer (Brinkmann, Westbury, NY), and total RNA was extracted from the snap-frozen salivary glands by the use of RNeasy Mini Kit (Qiagen; distributed through VWR International, Oslo, Norway). The manufacturer’s instructions were followed for RNA isolation from tissue, the RNA was eluted in 50 µL of RNase-free water, then 2 µL of white glycogen (Ambion Europe, Cambridge, UK), 10 µL of 5 M ammonium acetate (Ambion), and 150 µL of 96% ethanol were added. The mixture was left to precipitate at − 20 °C for 1 h. After centrifugation for 30 min at 4 °C utilizing the cooling centrifuge, the pellet was resuspended in RNA storage solution (Ambion). The optical density and yield were measured in a model 2100 Bioanalyzer from Agilent Technologies (Palo Alto, CA). Total RNA was preserved at − 80 °C until used. The total RNA used for the microarray analyses and the quantitative RT-PCR was from the same batches of RNA^35^. The primer sequences used for quantitative RT-PCR of the studied genes namely; superoxide dismutase 1 (SOD 1), catalase (CAT), aquaporin 5 (AQP 5) and alpha amylase 1 A (AMY 1 A), are represented in (Table 2).

**Table 2:** The primer gene sequences used for quantitative RT-PCR.

Gene	Sequence
SOD 1	Forward CGTCATTCACTTCGAGCAGA Reverse AAAATGAGGTCCTGCAGTGG
CAT	Forward ACATGGTCTGGGACTTCTGG Reverse AAGGTGTGTGAGCCATAGCC
AMY 1 A	Forward CAGACAGCACTTGTGGCAAT Reverse ACCACATTCCTGAAGGCAAC
AQP 5	Forward GGGCCATCTTGTGGGGATCT Reverse CCAGTGAGAGGGGCTGAACC

Abbreviations: RT-PCR; real time-polymerase chain reaction, SOD 1; superoxide dismutase 1, CAT; catalase, AMY 1 A; alpha amylase 1 A, AQP 5; aquaporin 5.

#### Statistical analysis

In vitro and in vivo data were presented as mean ± standard deviation (SD). Statistical differences between the results were compared via applying one-way ANOVA followed by Tukey-Kramer or Dunnett’s multiple comparisons tests contingent on the comparisons. Such data processing and comparison were accomplished utilizing GraphPad Prism^®^ V 5.00 (GraphPad Software, Inc., La Jolla, CA, USA). RT-PCR data and caspase-3 expression levels were analyzed via IBM SPSS software package V 20.0. (Armonk, NY: IBM Corp). The *P*-values were judged to be statistically significant at the threshold of *P* ≤ 0.05.

## Results and discussion

### Preparation and characterization of Chr-loaded PLGA NPs

In that investigation, all Chr-loaded PLGA NPs were prepared efficiently by double emulsion/solvent evaporation technique, where the internal aqueous phase (w_1_) was kept up invariable at 0.5% w/v PVA (2.5 mL) in all formulae. Yet, in the organic phase (o), different weight ratios of Chr and PLGA were added; besides, in the outer aqueous phase (w_2_), different PVA concentrations were utilized. Then, DEE %, D_p_, PI and ζP, as the pursuing criteria, were assessed to clarify the effect of such variables on NPs properties and to select the most appropriate nanoparticulate system with respect to the measured criteria (Table 3).

**Table 3:** Properties of Chr-loaded PLGA NPs formulations.

Formula code	DEE %	D_*p*_ (nm)	PI	ζP (mV)
F1	99.71 ± 0.04	316.00 ± 07.21	0.166 ± 0.03	-20.23 ± 0.12
F2	99.89 ± 0.06	352.70 ± 14.00	0.475 ± 0.02	-15.70 ± 0.90
F3	99.62 ± 0.16	340.63 ± 27.41	0.408 ± 0.19	-14.73 ± 0.32
F4	99.98 ± 0.01	371.83 ± 07.96	0.123 ± 0.02	-16.67 ± 0.49

Each value represents the mean ± SD (*n* = 3).

Abbreviations: Chr-loaded PLGA NPs; chrysin-loaded poly(d, l-lactide-co-glycolide) nanoparticles, DEE %; drug entrapment efficiency, D_p;_ particle diameter, PI; polydispersity index, ζP; Zeta potential, SD; standard deviation.

### DEE %

In fact, the delivery of the recommended therapeutic dosage with the least amount of NPs formulation is a necessity to reduce the cost of the used materials, besides the production process, thence increasing EE % is the perfect endeavor to achieve such goal.

As evinced in Table 3, high values of DEE % (> 99%) for all Chr-loaded PLGA NPs was achieved, which could be explained based on the peculiarities of both the drug and the carrier. Naturally, Chr, being a lipophilic material, can be efficiently encapsulated by PLGA^21,36,37^.

### D_p_, PI and ζP

Fundamentally, intracellular uptake of NPs in mucosal and epithelial tissues is determined based on D_p_ with its subsequent effect on the therapeutic efficiency^38^. All the prepared Chr-loaded PLGA NPs formulations disclosed small D_p_ values ranging from 316 ± 7.21 to 371.83 ± 7.96 nm (Table 3).

Conspicuously, formulations containing high PLGA ratio (F2 and F4) exhibited a slight increase in the D_p_, compared to the ones with lesser PLGA ratio (F1 and F3), that could be ascribed to the increase in the viscosity of the organic phase (o) with the consequent net shear stress decrease and the simultaneous larger particles production. Such results resemble earlier studies^39^.

PI values of the developed Chr-loaded PLGA NPs formulations (Table 3), as an important tool for determining the breadth of size distribution, ranged from 0.166 ± 0.03 to 0.475 ± 0.02, stating the NPs’ homogeneous and uniform size distribution.

ζP, as an indicator of surface charge and colloidal stability, is influenced by surface chemistry of the formulae components. Herein, for all formulae, ζP values varied slightly between − 14.73 ± 0.32 and − 20.23 ± 0.115 mV (Table 3). Predominantly, such negative values probably due to the free carboxylic group on the PLGA polymer’s surface after the involvement of the carboxylic functional groups in hydrogen bond formation with phenolic hydroxyl ones of Chr. Comparable other drug loaded in PLGA NPs is in a fair agreement with the experimental results^40^.

The antecedent results, along with all valuable attained properties that are required in NPs, vindicated that Chr-loaded PLGA NPs formulation (F1) was the chosen formula with regard to high EE % of 99.71 ± 0.04%, minimum D_p_ of 316 ± 7.21 nm, small PI of 0.166 ± 0.03 as well as negative ζP of -20.23 ± 0.12 mV (Table 3), hence, it was subjected to further investigations.

### Characterization of the chosen formula

#### Morphological analysis

**Fig. 2:**
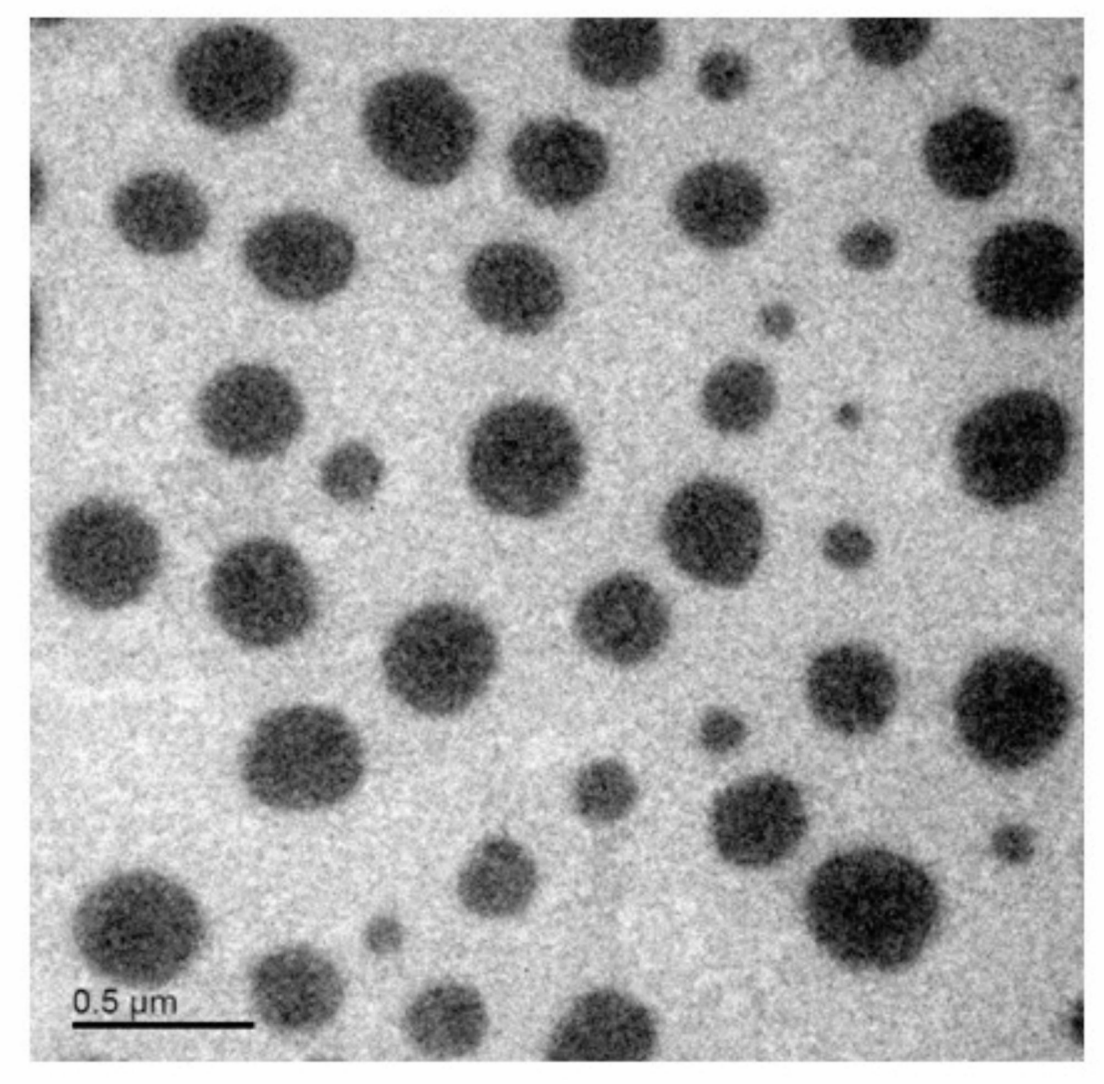
TEM photograph of the chosen Chr-loaded PLGA NPs (F1). Abbreviations: TEM; transmission electron microscopy, Chr-loaded PLGA NPs; chrysin-loaded poly(d, l-lactide-co-glycolide) nanoparticles.

Figure 2 shows the TEM photograph of the chosen Chr-loaded PLGA NPs (F1). It emerged spherical, nanoscopic, non-assembled particles, jointly with a smooth surface. Similar findings have been reported with other PLGA NPs^41,42^.

#### FT-IR spectroscopic analysis

**Fig. 3:**
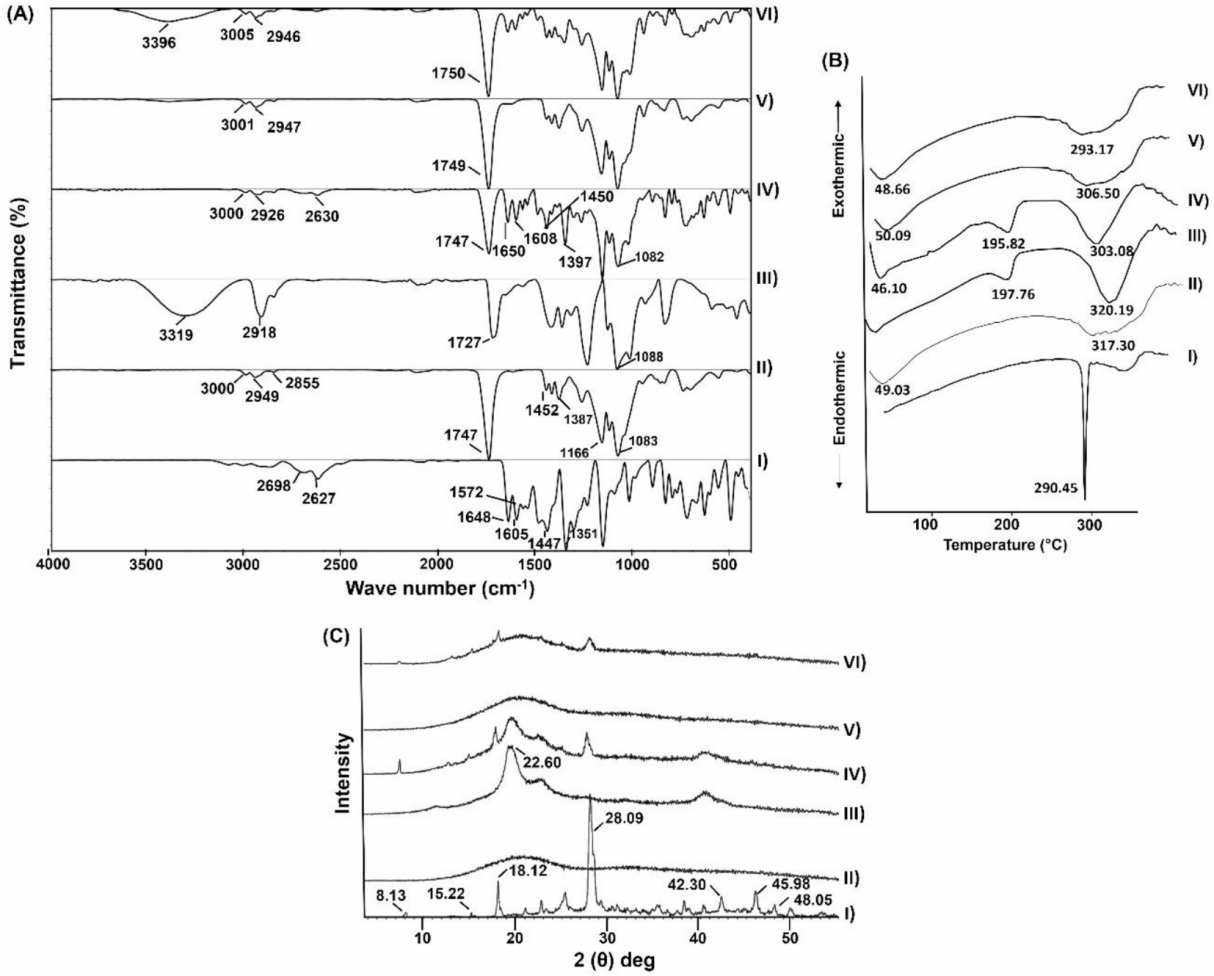
(**A**) FT-IR spectra, (**B**) DSC thermograms, and (**C**) XRD patterns of Chr (I), PLGA (II), PVA (III), PM of Chr, PLGA, and PVA (IV), blank PLGA NPs (V) and Chr-loaded PLGA NPs (F1) (VI). Abbreviations: FT-IR; Fourier transform-infrared spectroscopy, DSC; Differential scanning calorimetry, XRD; X-ray diffraction, Chr; chrysin, PLGA; poly(d, l-lactide-co-glycolide), PVA; polyvinyl alcohol, PM; physical mixture, PLGA NPs; poly(d, l-lactide-co-glycolide) nanoparticles, Chr-loaded PLGA NPs; chrysin-loaded poly(d, l-lactide-co-glycolide) nanoparticles.

The infrared spectra of the chosen Chr-loaded PLGA NPs (F1) and its constituents were depicted in Fig. 3A. The FT-IR spectrum of Chr (I) exhibited the bands of all its distinguishing functional groups at 2698 and 2627 cm^− 1^ for the (C–H) stretching band and a peak at 1648 cm^− 1^ for (C=O) stretching. Besides, the characteristic bands at 1605, 1572, and 1447 cm^− 1^ originated from (C=C) stretching. Moreover, the band at 1351 cm^− 1^ was correlated to the (C–O) stretching^28^. The PLGA infrared spectrum (II) demonstrated a discriminatory peak located at 3000 and 1747 cm^− 1^ were assigned to the (–OH) and (C=O) stretching vibrations of the fundamental monomers “lactide and glycolide.” Furthermore, the two differentiated infrared shoulders at 2949 and 2855 cm^− 1^ were ascribed to (–CH_3_, –CH_2_ and –CH) symmetric stretching. The peaks at 1452 and 1387 cm^− 1^ were correlated to asymmetric stretching of –CH_2_ as well as –CH_3_, respectively, whereas those around 1166–1083 cm^− 1^ proposed stretching (C–O) vibration^42–44^. In the infrared spectra of PVA (III), the featured peak at 3319 cm^− 1^ was owing to stretching vibrations of the (–OH) group and the intermolecular and intramolecular hydrogen bonds. The three special bands allocated at 2918, 1727 and 1088 cm^− 1^ emanated from stretching vibrations of (C–H, C=O and C–O–C), respectively^45,46^. The PM spectra (IV) displayed the infrared shoulders of F1 individual constituents namely; Chr, PLGA, and PVA. The spectra of blank (V) and medicated (VI) freeze-dried Chr-loaded PLGA NPs (F1) synchronized together, besides the broadening of the PVA characteristic band in the medicated NPs spectrum, which correlated to the probability of hydrogen bonding among its hydrophilic groups and Chr’s (-OH) phenolic groups. Interestingly, Chr’s characteristic bands were invisible in the medicated NPs spectrum. These alterations reflect Chr entrapment in the NPs’ polymeric matrix^47^.

#### DSC analysis

A strongly melting endothermic peak occurred at 290.45 °C, which clearly revealed the Chr (I) crystalline nature, was illustrated in Fig. 3B^28,47^. Contrarily, in the DSC thermograms of both PLGA (II) and PVA (III), two broad endothermic peaks were delineated. The peaks correlated to the earmarked glass transition (Tg) and thermal decomposition temperatures of PLGA (II) were at around 49.03 and 317.30 °C, whereas those obviously recorded at 197.76 and 320.19 °C were ascribed to the melting degree as well as structural decomposition of PVA (III), respectively^42^.

The distinct peaks of the preceding two polymers with a conspicuous lack of those regarding Chr, owing to the dilution impact, were discerned at their particular positions in the PM (IV) thermogram. The thermogram of medicated freeze-dried Chr-loaded PLGA NPs (F1) (VI) was consistent with that of the blank one (V), along with the subsistence of conspicuous PLGA native peaks with minor shifting. Furthermore, the absence of indigenous Chr’s peak in the thermogram of medicated NPs (VI) indicates the loss of Chr’s crystalline nature and its successful encapsulation in the polymeric matrix. These results are consistent with the FT-IR data and are in substantial agreement with a previously substantiated one^47^.

#### XRD analysis

The XRD diffractograms of the chosen Chr-loaded PLGA NPs (F1) and its constituents were represented in Fig. 3C Sharp diffraction peaks of Chr (I) at 2θ of 8.13°, 15.22°, 18.12°, 28.09°, 42.30°, 45.98°, and 48.05°, evidenced its crystallinity^48–50^. Contradictory, XRD diffractogram of PLGA (II) showed no distinctive peaks demonstrating its amorphous nature. Such results in consonance with previously documented ones^51^. A semi-crystalline structure of PVA (III) was stipulated by a broad diffraction peak at 2θ of 22.60° where its hydroxyl groups form both intra- and intermolecular hydrogen bonds^42^. The diffraction peaks of the ingredients of the investigated Chr-loaded PLGA NPs (F1) were identified at their specific positions in the PM (IV) diffractogram. Notably, the XRD patterns of blank (V) and medicated (VI) freeze-dried Chr-loaded PLGA NPs (F1) were consistent with one another, with a considerable expanding of the PVA diffraction pattern. Surprisingly, the medicated (VI) freeze-dried Chr-loaded PLGA NPs (F1) diffractogram showed a substantial reduction in the typical diffraction peaks intensity of Chr, suggesting a change in Chr’s crystalline nature upon encapsulation in the PLGA NPs matrix. As a consequence, the XRD findings manifested Chr’s incorporation into PLGA matrix in an amorphous distribution, as observed in the DSC analysis. Such behaviors had already been recognized^47^.

#### In vitro release study

**Fig. 4:**
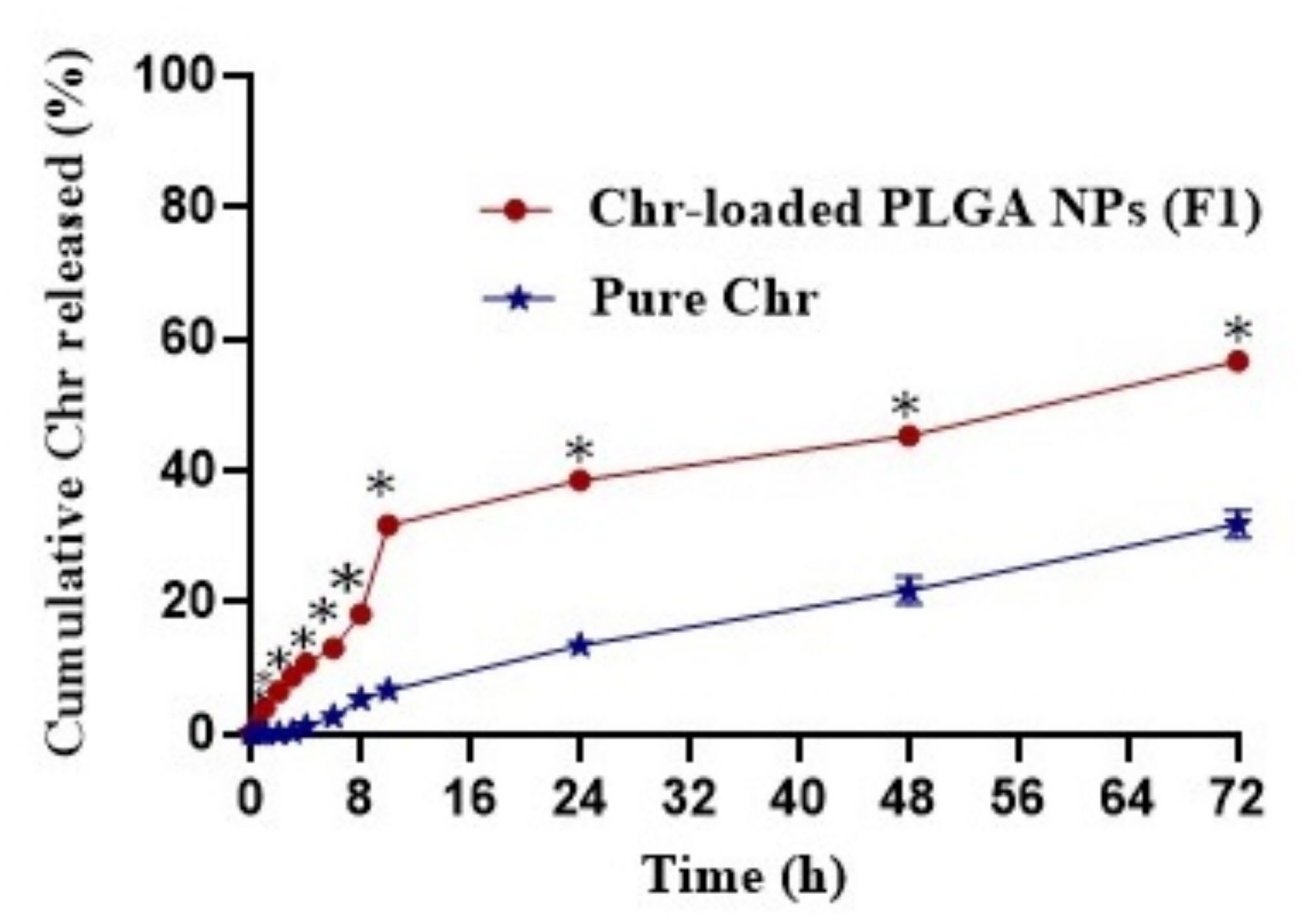
The in vitro release pattern of Chr from the chosen Chr-loaded PLGA NPs (F1) in comparison with its diffusion from aqueous suspension at pH 7.4. Each point represents the mean ± SD (*n* = 3). ^*^ Significant at *p* < 0.05 Chr-loaded PLGA NPs formula (F1) versus drug suspension. Abbreviations: Chr; chrysin, Chr-loaded PLGA NPs; chrysin-loaded poly(d, l-lactide-co-glycolide) nanoparticles, SD; standard deviation.

The in vitro drug release studies are the important for confirming the vehicle’s ability to release the medication and for gaining insight into the rate at which this phenomenon transpires. As evidenced in Fig. 4, the diffusion profile of Chr from an aqueous suspension as compared to its in vitro release one from the chosen Chr-loaded PLGA NPs (F1) was studied at pH 7.4 to mimic the physiological pH of the blood. Chr suspension exhibited a lag time of around 2 h, which might be ascribed to its lipophilic nature. Additionally, by the end of the experiment the percentage released of Chr from its suspension was only 31.84% ± 2.12. Comparatively, the percentage released of Chr from the selected Chr-loaded PLGA NPs formula (F1) reached 56.59% ± 0.81 after 72 h. As illustrated in Fig. 4, the release of Chr from (F1) acquired a sustained release behavior. As beforehand reported, drug release from PLGA NPs embodies the combined consequences of pore diffusion, surface erosion, in situ cross-linking and degradation processes^42,52^. Interestingly, statistical analysis, using one-way ANOVA test revealed a significant difference (*P* < 0.05) between the percentage release of Chr from Chr-loaded PLGA NPs (F1) and its diffusion from an aqueous suspension after 1 h till the end of the experiment (Fig. 4). Inclusively, the above results endorsed the superiority of the chosen Chr-loaded PLGA NPs formula (F1) as a nanocarrier for Chr and revealed its encapsulation within the NPs’ polymeric matrix in an amorphous state, triggering a higher solubility and an improved Chr release rate.

### Kinetic analysis of Chr release data

Drug release kinetics is a substantial discipline of pharmaceutical studies that explores the time-dependent behavior of pharmaceuticals after delivery. It was disclosed that pure Chr diffusion exhibited exceptional characteristics with an impressive R^2^ value of 0.986. Conspicuously, the first-order kinetics outperformed all other models investigated in this study (as shown in Table 4). On the other hand, when investigating the chosen Chr-loaded PLGA NPs formula (F1), we noticed an impressive R^2^ value of 0.908, but with Higuchi model dominance overall. Further complementary analysis by the Korsmeyer-Peppas empirical equation established a non-Fickian diffusion mechanism (*n* > 0.5), indicating that the drug release from NPs was mainly governed by two phenomena: drug diffusion and polymeric matrix relaxation. The same kinetic behavior was followed by other comparable drugs loaded in PLGA NPs^53,54^.

**Table 4:** Coefficients of determination (R^2^) for kinetic analysis of the percentage drug diffused and that released from pure Chr as well as the chosen Chr-loaded PLGA NPs formula (F1), respectively.

Chr form	*R* ^2^			Korsmeyer-Peppas	
	Zero- order	First- order	Higuchi-diffusion	*R* ^2^	Diffusional exponent (*n*)
Pure Chr	0.981	0.986	0.949	-	-
Chr-loaded PLGA NPs formula (F1)	0.843	0.857	0.908	0.938	0.68

Abbreviations: Chr; chrysin, Chr-loaded PLGA NPs; chrysin-loaded poly(d, l-lactide-co-glycolide) nanoparticles, R^2^; coefficients of determination.

Stability study.

### Stability study

Table 5 illustrates the stability of the chosen Chr-loaded PLGA NPs formula (F1), designated as D_p_, PI, and ADR%. Auspicious outcomes for the assessed parameters were noted throughout the storage duration at 4 ± 1 °C, with no observed color or odor alterations. When compared to the values of the freshly made formula (Chr-loaded PLGA NPs formula (F1)) at zero time (*P* > 0.05), D_p_, PI, and ADR% were nearly unaffected. According to these results, the selected Chr-loaded PLGA NPs formula (F1) demonstrated excellent physical stability when refrigerated, confirming its effectiveness. Favorable results for the stability of PLGA NPs have been previously reported^32^.

**Table 5:** Stability assessment data of the chosen Chr-loaded PLGA NPs formula (**F1**) following storage at refrigerated temperature (4 ± 1 °C).

Time for storage	Evaluation parameters		
	Refrigerated temperature (4 ± 1 °C)		
	D_*p*_ (nm)	PI	ADR%
Initial	316 ± 7.21	0.166 ± 0.03	100.00 ± 0.00
1st month	326 ± 15.66	0.283 ± 0.07	99.72 ± 0.03
2nd month	334.10 ± 10.69	0.240 ± 0.03	99.44 ± 0.64
3rd month	343.30 ± 5.80	0.223 ± 0.01	99.09 ± 0.28

Each value represents the mean ± SD (*n* = 3).

Abbreviations: Chr-loaded PLGA NPs; chrysin-loaded poly(d, l-lactide-co-glycolide) nanoparticles, D_p;_ particle diameter, PI; polydispersity index, ADR%; average drug retention percent, SD; standard deviation.

### Effective counteraction of the chosen Chr-loaded PLGA NPs (F1) against cisplatin-induced toxicity in submandibular salivary glands in Albino rats

#### Histopathological examination

**Fig. 5:**
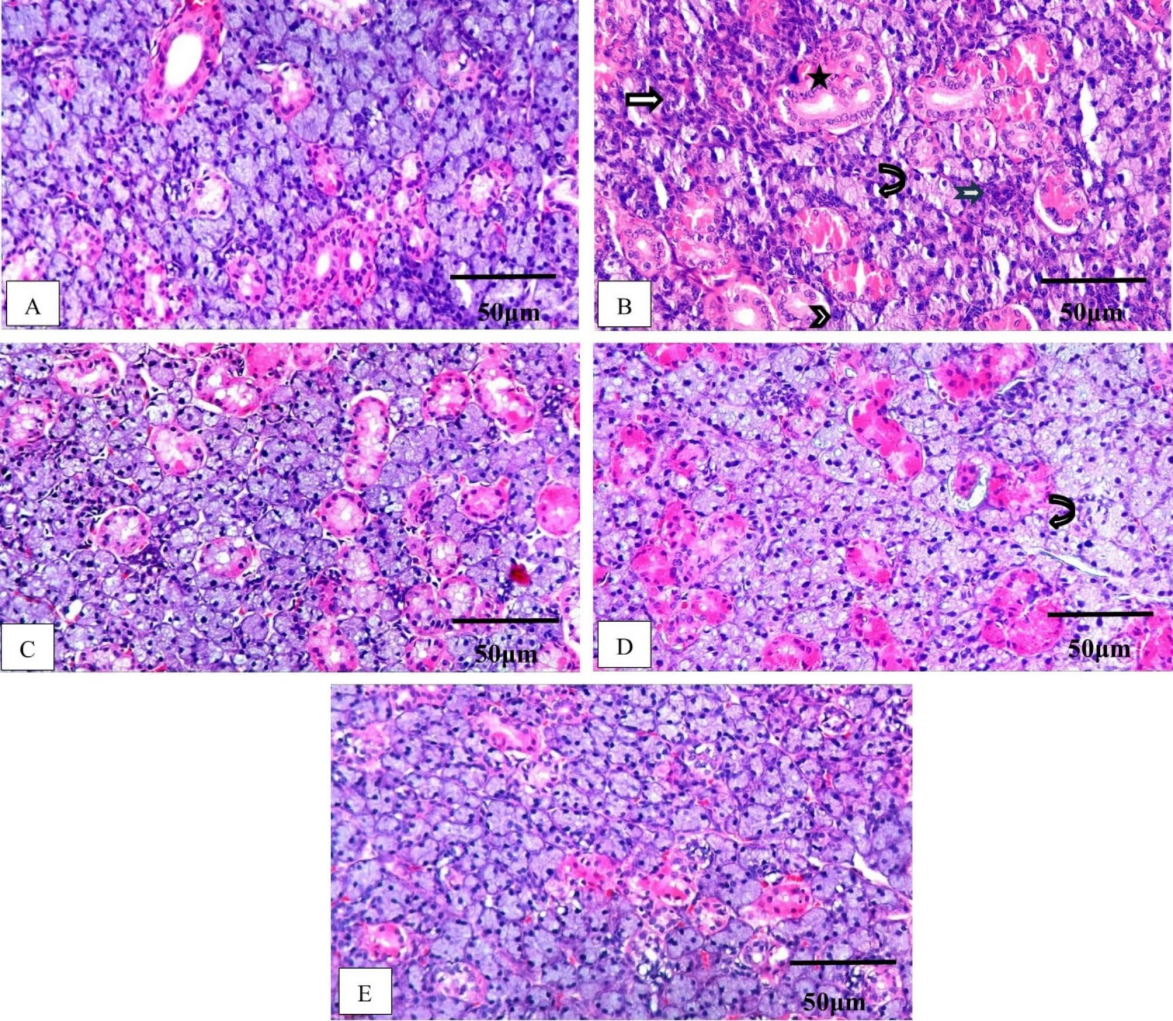
Photomicrographs of microscopical examination of rat submandibular glands sections stained by (H&E) (X: 200, Scale bar: 50 μm). (**A**) Negative control (N) group showed rounded serous acini with narrow lumen and duct structure, the nuclei appeared spherical and basally situated, (**B**) cisplatin-induced toxicity group revealed loss of acinar outline (arrow), vacuolization (curved arrow), large aberrant nuclei (arrowhead), apoptotic nuclei (notched arrow), and the duct shows a disrupted outline (star), (**C**) pure Chr group where the gland sections showed partial restoration of the acinar outline with nearly normal nuclear pattern (arrowhead), vacuolization was reduced, and the ductal outline was ameliorated, (**D**) blank NPs group showed marked vacuolization, the acinar and ductal outlines showed shrinkage (arrow), and (**E**) Chr-loaded PLGA NPs group almost restored normal structure in acini as well as ducts and showed minimal vacuolization. Abbreviations: H&E; hematoxylin and eosin, Chr; chrysin, NPs; nanoparticles, Chr-loaded PLGA NPs; chrysin-loaded poly(d, l-lactide-co-glycolide) nanoparticles.

Submandibular salivary glands of negative control (N) group showed a normal appearance of both acinar and ductal structures (Fig. 5A). In contrary, cisplatin-induced toxicity group manifested shrinkage of both serous acini and ducts with loss of architecture of the serous acini and granular convoluted tubules, marked vacuolation, pyknotic, and hyperchromatic nuclei (Fig. 5B). These findings may be attributed to accelerated apoptosis of acinar cells and degeneration caused by cisplatin^55^.

 Intra-cytoplasmic vacuoles may be present due to the released free radicals that cause damage to cellular components. Moreover, cellular changes such as hyperchromatism, irregular membrane outline, and pyknotic nuclei may result from nuclear damage and DNA platination which induce inter- and intrastrand DNA adducts^56^.

 The pure Chr group showed a restored structure of serous acini compared to the cisplatin-induced toxicity group with some vacuolation and reduced shrinkage of ducts Fig.  5C). These results may be explained as Chr was proved to restore the redox cycle components (glutathione reductase (GR), glutathione peroxidase (GPx), and reduced glutathione (GSH)). Additionally, it restored enzymatic CAT levels which are responsible for the breakdown of hydrogen peroxide into water and oxygen. Chr potentiated endogenous defense enzymes against cisplatin-induced oxidative stress in renal, jejunum, colon, and lung tissues in rats^57,58^.

 Blank NPs group results Fig.  5D) were in conjunction with the previous investigation which stated that PLGA microparticles were safely injected directly into the parotid gland tissue; they modified slightly morphometric parameters of the duct lumen and thickness of the acini without evoking tissue inflammatory response, despite the presence of polymer waste^59^.

 Intriguingly, Chr-loaded PLGA NPs group showed a nearly normal structure with a pyramidal shape of serous acinar cells surrounding a narrow lumen, with minimal vacuolation and restoration of duct structure Fig.  5E). Chr in pure form has poor solubility, rapid metabolism, and excretion. Contrariwise, Chr-loaded PLGA NPs showed enhanced bioavailability, increased solubility, a higher free radical scavenging activity, and reduced cytotoxicity. These findings agreed with a previous study showing the enhanced chemotherapeutic effect of Chr-loaded PLGA NPs in breast cancer T47D cells^60^. Another study presented that the nano-encapsulation of Chr has potentiated its antioxidant and anti-inflammatory activities and exhibited a protective effect against cadmium-induced toxicity in mice^61^.

### IHC assessments

The expression of caspase-3 was detected as brown staining of the cytoplasm of acini and ducts. Caspase-3 belongs to the cysteine protease family, which is responsible for breaking down essential cellular proteins involved in the characteristic morphological changes that occur in cells undergoing apoptosis^62^.

**Fig. 6:**
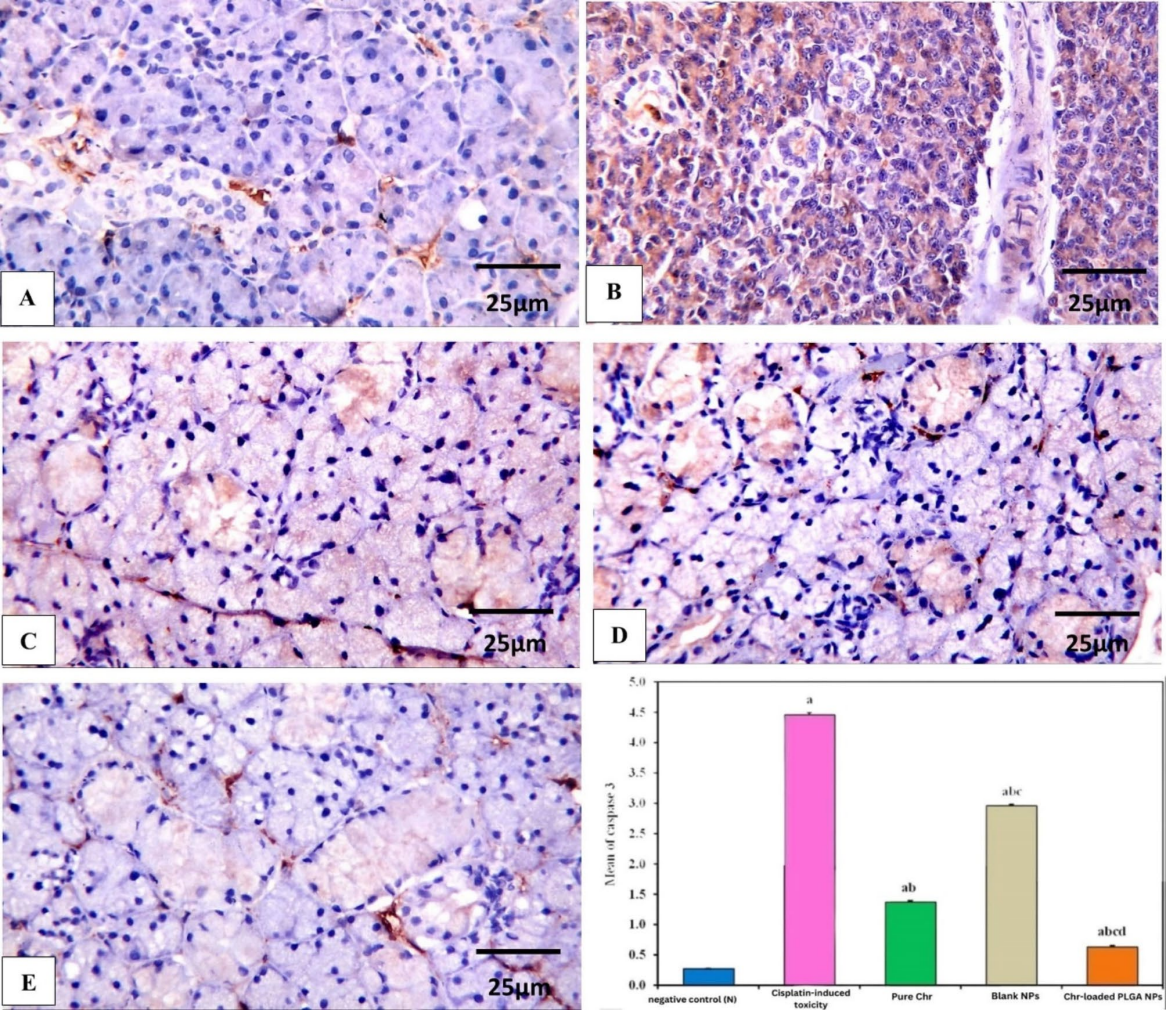
Photomicrographs of caspase-3-immunostained sections showing variable intensity of acinar and ductal structures with reaction being weak in negative control (N) group (**A**), cisplatin-induced toxicity group showed intense reaction (**B**), pure Chr and blank NPs groups revealed moderate reaction (**C**, **D**), while Chr-loaded PLGA NPs group showed a mild reaction (**E**), (X: 400, Scale bar: 25 μm). The chart bar shows levels of caspase-3 expression in all groups analyzed by one-way ANOVA, where a: significant with negative control (N) group, b: significant with cisplatin-induced toxicity group, c: significant with pure Chr group, d: significant with blank NPs group. Abbreviations: Chr; chrysin, NPs; nanoparticles, Chr-loaded PLGA NPs; chrysin-loaded poly(d, l-lactide-co-glycolide) nanoparticles, ANOVA; analysis of variance.

The negative control group revealed weak expression) 0.27 ± 0.005) (Fig. 6A), while the cisplatin-induced toxicity group showed severe expression of caspase-3 with mean and SD of 4.46 ± 0.033 (Fig. 6B). Badawy et al.^63^ concluded in their study that cisplatin causes DNA plastination and cytotoxic alterations that cause apoptosis with a significant increment in the area percentage of caspase-3 expression.

Moreover, pure Chr, blank NPs, and Chr-loaded PLGA NPs groups showed a significant decrease when compared to cisplatin-induced toxicity one with mean and SD (1.37 ± 0.024), (2.96 ± 0.025) (0.63 ± 0.023), respectively (Fig. 6C–E). These results were consolidated with Fagundes et al.^64^ who found that Chr ameliorated the healing of gastric lesions by reducing the expression of caspase-3 as well as matrix metalloproteinase (MMP-9) and El-Marasy et al.^65^ who proved that Chr and Chr-loaded nanovesicles decreased the elevated expression of caspase-3, in diabetic rats through activation of nerve growth factor/protein kinase B/glycogen synthase kinase 3β (NGF/AKT/GSK-3β) mediating potent anti-apoptotic effect.

### RT-PCR analysis

#### Effect of the chosen Chr-loaded PLGA NPs (F1) against cisplatin-induced oxidative stress in submandibular salivary glands

Since the oxidative stress is one of the destructive mechanisms of cisplatin on the submandibular salivary glands, the gene expression levels of SOD 1 and CAT were estimated in all investigated groups. As illustrated in Table 6, a substantial decrease was detected (*P* < 0.001) in the expression levels of both SOD 1 and CAT in cisplatin-induced toxicity group, compared to negative control one. These findings agree with Bomfin et al.^66^ who observed a significant reduction in the SOD and CAT levels in the saliva of rats 10 days after the first 5-fluorouracil injection. Moreover, other studies reported that tissue injury by cisplatin occurred due to the induction of oxidative stress in liver^67^, testicular tissue^68^ and kidney^69,70^. Nevertheless, a significant increase (*P* < 0.001) was noticed in the expression levels of SOD 1 and CAT in pure Chr group with respect to cisplatin-induced toxicity one. Previous studies illustrated that Chr increased the levels of GSH and enzymatic activities of CAT, GR, GPx, and glutathione-S-transferase in colon tissue of rats subjected to cisplatin-induced cytotoxicity^57^. Moreover, Chr counteracted the hepatic oxidative stress induced by methotrexate in rats^71^. Similar actions were also reported in doxorubicin and 5-fluorouracil animal models^72,73^. This powerful antioxidant effect of Chr is endorsed for scavenging the harmful free radicals owing to the hydroxyl substitution in the 5th and 7th positions that can sequestrate the free radicals^74^. Interestingly, the administration of Chr-loaded PLGA NPs displayed a substantial improvement (*P* < 0.001) in these genes compared to other groups. These results indicate that the Chr-loaded PLGA NPs evoked a higher antioxidant action than that of pure Chr. In consistence with these outcomes, similar previous study^75^ observed that Chr-loaded PLGA NPs counteracted the hippocampal oxidative stress in epileptic rats via upregulating the nuclear factor erythroid-2 related factor 2/antioxidant response element/heme oxygenase-1 (Nrf2/ARE/HO-1) pathway. Also, Chr-loaded PLGA NPs evoked a noticeable antioxidative impact in animal models of Alzheimer’s disease^76,77^. This indicates that the therapeutic efficacy and bioavailability of Chr could be increased by encapsulating Chr in PLGA scaffold.

**Table 6:** Influence of the chosen Chr-loaded PLGA NPs (F1) on cisplatin-induced oxidative stress in submandibular salivary glands instigated expression of SOD 1, CAT, AMY 1 A, and AQP 5 in comparison with different experimental groups using RT-PCR.

Animal groups	SOD 1	CAT	AMY 1 A	AQP 5
Negative control (N)	1 ± 0.04	1 ± 0.01	1 ± 0.04	1 ± 0.05
Cisplatin-induced toxicity	0.23^a^ ± 0.04	0.11 ± 0.02	0.12 ± 0.02	0.12 ± 0.01
Pure Chr	2.35^ab^ ± 0.29	4.95^ab^ ± 0.39	2.40^ab^ ± 0.01	3.41^ab^ ± 0.35
Blank NPs	1.79^ab^ ± 0.24	1.51^bc^ ± 0.07	1.99^b^ ± 0.14	1.47^bc^ ± 0.44
Chr-loaded PLGA NPs	3.41^abcd^ ± 0.13	7.11^abcd^ ± 0.31	6.75^abcd^ ± 0.73	9.65^abcd^ ± 0.19
F value	92.332^*^	345.778^*^	119.580^*^	416.503^*^
*P* value	< 0.001^*^	< 0.001^*^	< 0.001^*^	< 0.001^*^

Data are expressed as the mean ± SD (*n* = 3).

^*^Significant at *P* ≤ 0.05.

Statistical significances of animal groups are indicated as follows: a; significant with negative control group, b; significant with cisplatin-induced toxicity group, c; significant with pure Chr group, d; significant with blank NPs group.

Abbreviations: Chr-loaded PLGA NPs; chrysin-loaded poly(d, l-lactide-co-glycolide) nanoparticles, SOD 1; superoxide dismutase 1, CAT; catalase, AMY 1 A; alpha amylase 1 A, AQP 5; aquaporin 5, RT-PCR; real time-polymerase chain reaction, Chr; chrysin, NPs; nanoparticles, SD; standard deviation.

#### Effect of the chosen Chr-loaded PLGA NPs (F1) on salivary marker genes of submandibular salivary gland in cisplatin-challenged rats

The salivary-specific gene expressions, AMY 1 A and AQP 5, were determined to explore the effect of different drug forms on the function of salivary glands affected by cisplatin toxicity. Saliva has various oral functions, as moistening the mucosa and constantly protecting it from injury and bacterial infections. Aquaporins (AQPs) are proteins that can selectively control water transport across membranes. Among them, AQP 5 is a widely used marker to assess the drying effect of tested chemicals on salivary glands^78^. Also, AMY 1 A indicates the synthesis of proteins in the serous acinar cells of the salivary glands, which reflects the glandular cell damage, and its decrease may increase the risk of oral mucositis during chemoradiotherapy^79^. Excessive exposure to anticancer medications can cause apoptosis of glandular cells, a decrease in the total number of cells, and a weakening of secretory function, with a subsequent decrease in the amount of glandular secretions^79^. Xerostomia is the most common adverse effect of chemotherapy in patients suffering from head and neck cancer as a result of permanent salivary gland hypofunction^80^. According to previous investigation, stated that cisplatin and 5-fluorouracil caused a significant decrease in salivary gland weights^81^. The decline in AMY 1 A and AQP 5 secretion may be attributed to the increased free radicals and oxidative stress as well as the morphometric alterations in the salivary glands^82^. However, their expression was significantly increased (*P* < 0.001) in pure Chr group (orally administered) compared to cisplatin-induced toxicity one. Astonishingly, the expression of AMY 1 A and AQP 5 were increased in Chr-loaded PLGA NPs treated group than that detected in pure Chr one (Table 6).

Collectively, the aforesaid in vivo findings were in harmony with each other, supporting the impressive counteraction effect of the chosen Chr-loaded PLGA NPs (F1) against cisplatin-induced toxicity in submandibular salivary glands. Such distinguished efficacy probably correlated to several interrelated elements, which are as detailed; (1) Chr possesses anti-oxidant as well as anti-inflammatory properties, denoting its pharmacological impact on cisplatin-induced toxicity in submandibular salivary glands^61,74^; (2) Chr encapsulation within the NPs’ polymeric matrix in an amorphous state, triggering a higher solubility leading to higher bioavailability compared to pure Chr^83^; (3) since the inflammatory submandibular salivary glands have higher permeability, therefore, NPs can passively integrate and accumulate into their bodies via their nanoscale and enhanced permeability and retention (EPR) effect^84,85^; (4) PLGA NPs have been proven to encapsulate Chr and efficiently provide sustained in vitro release; (5) Chr-loaded PLGA NPs can deliver the functional form of Chr to cells and exert a more potent synergistic effect when compared to Chr in its free form. Subsequently, it can be concluded that the incorporation of Chr into a PNDS is regarded as an efficient paradigm for mitigation of submandibular salivary gland damage, apoptosis in gland cells, oxidative stress, and finally the inflammatory cascade. Additionally, it might be regarded as therapeutic treasure for chemotherapy patients.

## Conclusion

Chr’s lipophilicity leads to its low aqueous solubility and poor bioavailability. Thence, the main intent of this contemporary study was to encapsulate Chr, a naturally occurring flavone with multiple biological activities, in PLGA NPs to address this issue. Chr-loaded PLGA NPs were successfully prepared adopting a double emulsion/solvent evaporation technique (w_1_/o/w_2_) to mitigate cisplatin-induced toxicity in Albino rats’ submandibular salivary glands. Solid state characterization of the chosen formula (F1) via FT-IR, DSC, and XRD vindicated Chr entrapment inside the polymeric matrix. Furthermore, the TEM photograph revealed its nanoscopic, spherical, non-assembled particles, jointly with a smooth surface. Eminently, the in vitro release profiles endorsed the Chr encapsulation within the NPs’ polymeric matrix in an amorphous state, triggering a higher solubility and a higher Chr release rate compared to pure Chr. Indisputably, astonishing in vivo counteraction effectiveness against cisplatin-induced toxicity in submandibular salivary glands in Albino rats was manifested upon comparing Chr-loaded PLGA NPs (F1) treated group with pure Chr as well as blank NPs treated ones. Based on the antecedent results, it could be deduced that the chosen Chr-loaded PLGA NPs formula (F1) protruded as a promising phytomedicine paradigm that could be ratified therapeutically. However, some study limitations should be acknowledged, where, the in vivo experiments did not fully consider the toxicities that can appear in other organs. Hence, a future study is required to clarify the role of Chr-loaded PLGA NPs formula (F1) against cisplatin-induced toxicity in different organs.

## Data Availability

The datasets used and/or analyzed during the current study are available from the corresponding author on reasonable request.
